# On the Inhalation of the Vapor of Ether in Surgical Operations

**Published:** 1849-07

**Authors:** 


### Bibliographical Notices.

*On the Inhalation of the Vapor of Ether in Surgical Operations, containing a Description of the Various Stages of Etherization and its Results, &c.* by Dr. Snow, Churchill, London.

The above work appears to have been written by one who has had some experience in the administration of the vapor of ether, and who has also paid considerable attention to its effects.

The author agrees with MM. Louget and Floureus, in their views regarding the five degrees of etherization, and observes, "the various changes of feeling that a person may experience, whilst he still retains a correct consciousness of where he is, and what is occurring around him, and a capacity to direct his voluntary movements. In the second degree, the mental functions may be exercised, and voluntary actions performed, but in a disordered manner. In the third degree, there is no evidence of any mental function being exercised, and consequently no voluntary motions occur; but muscular contractions, in addition to those concerned in respiration, may sometimes take place as the effect of the ether, or of external impressions. In the fourth degree no movements are seen, except those of respiration, and they are incapable of being influenced by external impressions. In the fifth degree, (not witnessed in the human being,) the respiratory movements are more or less paralyzed and become difficult, feeble, or irregular."

The results which the author has given, go to show, that the vapor of ether, when inhaled, exerts no injurious effects over the success of surgical operations, and unquestionably shows an important fact, that out of twenty-six severe surgical operations, only five died, which is considerably lower than the average mortality.

Whether the ether was the immediate or exciting cause of death was not ascertained; but one important truth is ascertained, that of those operated on for lithotomy, not one died, precisely the same results attended the inhalation in the breast operations.

To those who are desirious of becoming proficient in the art of administering the vapor of ether, this work will be found useful in a practical point—but after all, the party must have hundreds of cases before he must consider himself competent to conduct protracted and difficult surgical operations. R.

